# Biofunctional Nanofibrous Substrate for Local TNF-Capturing as a Strategy to Control Inflammation in Arthritic Joints

**DOI:** 10.3390/nano9040567

**Published:** 2019-04-08

**Authors:** Elisa Bacelo, Marta Alves da Silva, Cristina Cunha, Susana Faria, Agostinho Carvalho, Rui L. Reis, Albino Martins, Nuno M. Neves

**Affiliations:** 1 3B’s Research Group, I3Bs—Research Institute of Biomaterials, Biodegradables and Biomimetics, Headquarters of the European Institute of Excellence on Tissue Engineering and Regenerative Medicine, AvePark—Parque de Ciência e Tecnologia, Zona Industrial da Gandra, University of Minho, Barco, 4805-017 Guimarães, Portugal; elisabacelo@live.com.pt (E.B.); estranged28@gmail.com (M.A.d.S.); rgreis@i3bs.uminho.pt (R.L.R.); nuno@i3bs.uminho.pt (N.M.N.); 2 ICVS/3B’s—PT Government Associate Laboratory, Barco, 4805-017 Guimarães, Portugal; cristinacunha@med.uminho.pt (C.C.); agostinhocarvalho@med.uminho.pt (A.C.); 3 Life and Health Sciences Research Institute, Scholl of Medicine, Campus of Gualtar, University of Minho, 4710-057 Braga, Portugal; 4 Department of Mathematics for Science and Technology Research CMAT, Campus of Azurém, University of Minho, 4800-058 Guimarães, Portugal; sfaria@math.uminho.pt; 5 The Discoveries Centre for Regenerative and Precision Medicine, Headquarters at University of Minho, Avepark, Barco, 4805-017 Guimarães, Portugal

**Keywords:** antibody immobilization, electrospun nanofibers, TNF-α capture, human articular chondrocytes, rheumatoid arthritis

## Abstract

Rheumatoid arthritis (RA) is an autoimmune disease that affects the synovial cavity of joints, and its pathogenesis is associated with an increased expression of pro-inflammatory cytokines, namely tumour necrosis factor-alpha (TNF-α). It has been clinically shown to have an adequate response to systemic administration of TNF-α inhibitors, although with many shortcomings. To overcome such limitations, the immobilization of a TNF-α antibody on a nanofibrous substrate to promote a localized action is herein proposed. By using this approach, the antibody has its maximum therapeutic efficacy and a prolonged therapeutic benefit, avoiding the systemic side-effects associated with conventional biological agents’ therapies. To technically achieve such a purpose, the surface of electrospun nanofibers is initially activated and functionalized, allowing TNF-α antibody immobilization at a maximum concentration of 6 µg/mL. Experimental results evidence that the biofunctionalized nanofibrous substrate is effective in achieving a sustained capture of soluble TNF-α over time. Moreover, cell biology assays demonstrate that this system has no deleterious effect over human articular chondrocytes metabolism and activity. Therefore, the developed TNF-capturing system may represent a potential therapeutic approach for the local management of severely affected joints.

## 1. Introduction

Rheumatoid arthritis (RA) is an autoimmune disease that affects primarily the synovial cavity tissues on the small diarthrodial joints of the hands and feet [1], leading to persistent synovial inflammation [2] and progressive erosion of the articular structures [3,4]. The triggers for the disease susceptibility and the pathological cascade of events encompass environmental, genetic and stochastic factors [3,4]. For example, RA has special incidence on females [5], which is related with genetic [5,6,7] and hormonal factors [5,8,9,10], but also presents a heterogeneous geographic distribution, being less common in developing countries [11,12], which confirms the involvement of environmental and socio-cultural factors [12].

The disease pathophysiology involves several interconnected mechanisms. Specific antibodies for immunoglobulin G (IgG) mediate the autoimmune process, as well as the imbalanced expression of the pro-inflammatory cytokine’s profile and its functionality [1,12,13,14]. Consequently, this leads to inflammatory processes, autoimmunity enhancement, long-term inflammatory synovitis and joint damage [13,15]. Additionally, locally expressed degradative enzymes digest the cartilaginous matrix and destroy the articular surfaces [1]. Infiltration of B cells, CD4+ T cells and macrophages into the synovium, which in normal conditions is relatively acellular [1,14,16], leads to soft tissue oedema and stiffness [2]. Moreover, other inflammatory cells such as neutrophils, natural killer cells and mast cells play key roles in the disease’s progression [13].

From the autoimmune disease (AD) subsets that entail the multiple inflammatory cascades, one of the most significant players is the TNF-α, particularly the over-expression of the TNF-α [11,13,17]. TNF-α, a 233 amino acid protein [18], is a key signaling cytokine in the immune system mainly produced by monocytes, macrophages [19], and B and T cells [2,13,17]. TNF-α stimulates the production of other inflammatory mediators, namely IL’s, as well as the recruitment of immune and inflammatory cells into the joint [20,21]. As a regulatory cytokine that manages communication between immune cells and controls many of their functions when deregulated, TNF-α plays a key role in the pathogenesis of chronic inflammatory diseases, such as RA [22,23]. TNF deregulation in RA is linked to TNF-α converting enzyme (TACE or ADAM17), a metalloproteinase that cleaves trans-membrane TNF, releasing the soluble segment [18,24]; and to TNF receptors (mostly TNFr-I) [18,25,26], which are likely to be related with proinflammatory, cytotoxic and apoptotic responses [2].

The increasing knowledge on RA pathogenesis stimulated the development of different therapeutic modalities aiming to avoid joint destruction, minimize the symptomatic profile, and enhance physical function [27]. These options comprise analgesics, symptomatic management and inflammatory drugs [11,28]. Analgesics enclose the non-steroidal anti-inflammatory drugs (NSAIDs) that are commonly used to treat RA. The inflammatory suppressive drugs include glucocorticoids and disease-modifying anti-rheumatic drugs (DMARDs) both non-biologic and biologic. Due to the disease heterogeneity, therapeutic strategies must be tailored to the individual patient in order to achieve a low level of disease activity within a limited period of time [29]. Thus, the combination of two treatment modalities has been proposed to achieve such a purpose [30].

Considering the significant involvement of TNF-α in RA, as well as the rising evidences that this cytokine heads the pro-inflammatory cytokine cascade, it becomes a significant therapeutic target [31,32]. Specifically, these evidences led to the development of TNF inhibitors for the treatment of ADs [33,34,35,36,37], as they became the first class of biologic agents to be used in RA treatment [38]. A specific high affinity monoclonal antibody is used to recognize and neutralize selectively its antigen, i.e., TNF-α [18,27,39]. Five different types of TNF-α inhibitors are currently licensed for human clinical use in RA treatment, namely Infliximab, Entarnecept, Adalimumab, Certalizumab Pegol and Golimumab. With the exception of Infliximab, which is administrated by intravenous (IV) infusion, all other medicines are administered subcutaneously [13]. For long-term control of RA, a continuous treatment is required because of disease flares’ risk when the therapy is discontinued [1]. Although these therapeutic modalities have been used for quite some time, they present many shortcomings such as lack of specificity, limited antibody half-life, high cost, response variability, or even lack of response to treatments [11,40]. Due to the systemic character of these treatments, not only target tissues but also healthy tissues are exposed to a significant dose of drug, leading to adverse side effects [41]. These effects are common to many tissues, organs and systems, such as cardiovascular, renal, dermatological, and neurological, or risk of severe infection [11,27,28,40].

Furthermore, some attention has been given to drug carriers, in order to maintain effective concentration levels in plasma for extended periods [41]. Examples of these systems are the encapsulation of Infliximab in polylactide-co-glycolide microspheres [42] or porous silicon 3D structures [43], as well as long-term release of Etanercept by polyelectrolyte complex formulated particles [44]. Despite the promising preliminary results, these systems are still poorly developed.

Considering the shortcomings of these treatments, there is a need for innovative approaches that circumvent the aforementioned adverse side effects. Therefore, the main objective of this work was to develop an implantable system capable of capturing excessive TNF-alpha present in intra-articular cavities of an RA patient. For that, we immobilized a neutralizing TNF-α antibody at the surface of a polymeric substrate (i.e., electrospun nanofibers). This strategy takes advantage of specific interactions between the antibody and the antigen, where the antibody (TNF-α antibody) binds to the antigen (TNF-α) avoiding/limiting its harmful pro-inflammatory effects. After the system´s assembly and proved ability to capture TNF-α secreted by activated monocyte-derived macrophages, the cytotoxicity of the biofunctionalized system was tested with human articular chondrocytes. Indeed, the cytocompatibility and chondrogenic differentiation potential of bare electrospun nanofibers were previously reported by us [45,46,47], corroborating their potential use in cartilage regeneration approaches.

## 2. Materials and Methods

### 2.1. Materials

Polycaprolactone (PCL; Mn = 70,000–90,000 determined by Gas Permeation Chromatography), chloroform, *N*,*N*-dimethylformamide (DMF), 1,6-hexamethylenediamine (HMD), Ellmans reagent (DTNB), 2-iminiothiolane (2IT), 2-(*N*-morpholino)-ethanesulfonic acid (MES hydrate), phosphate buffered saline (PBS), *N*-hydroxysuccinimide (NHS), and 1-[3-(dimethylamino)propyl]-3-ethylcarbodiimide hydrochloride (EDC) were purchased from Sigma-Aldrich (Saint Louis, MO, USA) and kept at room temperature (RT), with the exception of 2IT which was kept at 4 °C and EDC which was kept at −20 °C. 4-(dimethylamino) pyridine (DMAP) was purchased from Millipore (Darmstadt, Germany) and kept at RT. Anti-TNF-α antibody [B-C7] was purchased from Abcam (Cambridge, UK) and kept at 4 °C until further use. Alexa Fluor^®^ 488 goat anti-mouse rabbit IgG (H + L) and TNF-α human ELISA kit were purchased from Life Technologies (Carlsbad, CA, USA) and kept at 4 °C until further use.

### 2.2. Production and Functionalization of Nanofiber Meshes

#### 2.2.1. Production of Nanofiber Meshes

Nanofiber meshes (NFMs) were prepared as described elsewhere [48,49]. Briefly, the NFMs were produced by electrospinning of a polymeric solution of 15% (*w*/*v*) PCL dissolved in an organic solvent mixture of chloroform and dimethylformamide (7:3 ratio). This PCL solution was electrospun by applying a voltage of 12 kV, a needle tip-to-ground collector distance of 20 cm and a flow rate of 1 mL/h. After the complete processing of 1 mL of PCL solution, the NFMs were left to dry for 1 day at RT inside a chemical hood, in order to completely evaporate the solvents. All further tests described were performed in samples of 10-mm squares.

#### 2.2.2. Surface Functionalization of Electrospun NFMs

The surface functionalization of electrospun NFMs was performed as described elsewhere [48,49]. Briefly, the NFMs’ surface was activated by 4 min of UV-Ozone irradiation (UV-O-Cleaner^®^, ProCleaner 220, Bioforce Nanoscience, Salt Lake City, UT, USA). Afterwards, the amine groups (–NH_2_) were inserted by immersion of the irradiated NFM in a 1 M hexanediamine (HMD) solution during 1 hour at 37 °C.

#### 2.2.3. Quantification of Amine Groups Present at the Functionalized NFMs

Quantification of amine groups present in each NFM was performed as described elsewhere [50,51]. For that, SH groups were inserted at the surface of the aminolysis-treated NFMs through the reaction of the amine groups with a 20 mM 2IT solution (0.1 mM PBS at pH8) and with a 20 mM DMAP solution during 1 h at 37 °C. Ellman’s reagent method was used to quantify the amino groups. For that, samples were immersed in 0.1 mM DTNB solution (in PBS 0.1 mM at pH 7.27) and incubated during 1 h at 37 °C. The absorbance of supernatants was measured in triplicate at 412 nm in a quartz plate, using the DTNB solution as blank. For the calculation of the 2-nitro-5-thiobenzoate (NTB^−2^) molar absorption coefficient, the value of 14,151 M^−1^ cm^−1^ was used [50,51].

### 2.3. Antibody Immobilization

The TNF-α antibody was immobilized at the surface of activated and functionalized electrospun NFMs. To determine the substrate’s maximum immobilization capacity, a wide range of primary antibody concentrations were considered (from 0 to 12 µg/mL). The TNF-α antibody was firstly activated by a 15-minute incubation with a solution of EDC/NHS (50 mM EDC and 200 mM NHS), dissolved in a 0.1 M 2-(*N*-morpholino)-ethanesulfonic acid (MES) buffer with 0.9% (*w*/*w*) NaCl, followed by pH adjustment to 4.7 [48].

The functionalized NFMs were incubated with 200 μL of the activated TNF-α antibody overnight at 4 °C. Biofunctionalized NFMs were washed three times with PBS. A 3% bovine serum albumin (BSA) solution was used as a blocking step. To determine the degree of TNF-α antibody immobilization, biofunctionalized NFMs were incubated with an Alexa Fluor^®^ 488 solution diluted in PBS (1:200 ratio). As a negative control, NFMs with no antibody immobilized (plotted as 0 µg/mL) were used. The fluorescence of the supernatant was determined in triplicate, using an excitation of 495/20 nm and an emission of 519/20 nm, and used to quantify the amount of antibody effectively immobilized.

### 2.4. Characterization Biofunctionalized NFMs

#### 2.4.1. Scanning Electron Microscopy (SEM)

SEM was used to analyse the morphology of biofunctionalized NFMs. Briefly, the biofunctionalized NFMs were sputter-coated with a thin layer (9–12 nm) of gold/palladium (Cressington 208 HR) and then analysed by SEM (NanoSEM, Nova 200, FEI company, Hillsboro, OR, USA). Micrographs were recorded at 15 kV with magnifications ranging from 100 to 2000 times.

#### 2.4.2. Fluorescence Microscopy

A fluorescence microscope (Axio Imager Z1m, Zeiss, Gottingen, Germany) was used to analyze the spatial distribution of the TNF-α antibody at the surface of electrospun NFMs. The TNF-α antibody was linked by a secondary antibody with green fluorescence (Alexa Fluor™ 488). Photographs were recorded at magnifications of 50, 200 and 400 times.

### 2.5. Capturing of TNF-α Present in Conditioned Medium of Macrophage Culture

A human monocytic cell line THP-1 was maintained in RPMI 1640 media supplemented with 2 mM L-glutamine, 100 µg/mL of penicillin, 100 μg/mL of streptomycin, 10 mM HEPES, and 10% fetal bovine serum (complete RPMI, cRPMI) (Life Technologies, Carlsbad, CA, USA). For the induction of cell differentiation, cells (106 per mL) were seeded in cRPMI with 100 nM phorbol 12-myristate-13-acetate (PMA) for 24 h. After incubation, non-attached cells were removed by aspiration, and the adherent cells were washed three times with cRPMI. To ensure reversion of cells to a resting macrophage phenotype before stimulation, cells were incubated for an additional 48 h in cRPMI without PMA. For stimulation and retrieval of conditioned media, cells were further incubated for 4 h with 100 ng/mL of lipopolysaccharide (LPS) in fresh cRPMI and the supernatants were collected and stored at −80 °C. Production of TNF-α was assessed in the conditioned media by commercial ELISA (Life Technologies, Carlsbad, CA, USA). Four systems were tested at least in quadruplicates, in two independent assays: (1) the biofunctionalized NFM (with immobilized TNF-α antibody); (2) a positive control with the soluble TNF-α antibody; (3) a negative control with the UV-O activated NFM; and (4) the conditioned cell culture medium alone. Each system was placed in sterile Eppendorf tubes and 2 mL of monocyte-derived macrophage conditioned medium was added individually, and kept at 37 °C in agitation in an orbital shaker at 120 rpm. Initially, a 3 days assay was performed with a 1 ng/mL TNF-α concentration from monocyte-derived macrophage conditioned medium. A specimen was collected at the following time points (2, 4, 6, 8, 10, 12, 24, 32, 48, and 72 h of incubation) without conditioned medium replacement. Afterwards, a 15-day assay was conducted, where a sample was collected daily and recharged with the same volume of recovered cell culture medium each day, which had a TNF-α concentration of 500 pg/mL. This concentration was chosen due to being closer to TNF-α levels in active RA patients (76.1 ± 103.2 pg/mL) [19], considering a margin of error affected by the TNF-α degradation.

#### Enzyme-Linked Immunosorbent Assay (ELISA)

For the quantification of TNF-α in the conditioned medium at each time point, sandwich TNF-α ELISA (KHC3012, TermoFisher Scientific, Carlsbad, CA, USA) was performed according to the manufacturer. Absorbance was read at 450 nm on a microplate reader (Synergy HT, BioTek, Winooski, VT, USA). A standard curve was built with concentrations ranging from 0 to 1000 pg/ml. The final concentrations were calculated by subtracting the value of each sample (i.e., testing conditions 1–3) to the conditioned culture medium baseline (i.e., testing condition 4), and the average was plotted for each testing condition along time.

### 2.6. Biological Assays

#### 2.6.1. Isolation and Cell Culture

Cartilage tissue consists of only one cell type, the chondrocyte, embedded in a dense extracellular matrix (ECM). To evaluate the cytotoxicity of the developed biofunctionalized nanofibrous substrates, human articular chondrocytes (hAC) isolated from knee cartilage samples, collected from arthroplasties surgeries biopsies, were used. These cells were chosen as they were isolated from diseased joints cartilage. Samples were collected under the cooperation agreement between Centro Hospitalar do Alto Ave, Guimarães, Portugal, and the 3B’s Research Group, after informed donor consent. Briefly, cells were isolated by enzymatic digestion, according to the protocol described previously [46].

Dulbecco’s modified Eagle’s medium (DMEM, Sigma D5671), containing 10 mM Hepes buffer (Life Technologies, Paisley, UK), l-alanyl-l-glutamine (Sigma), Non Essential Aminoacids (Sigma) 10,000 units/ml penicillin, 10,000 μg/mL streptomycin, and 10% foetal calf serum was the basis for the expansion and differentiation medium used herein (Basic medium). Basic medium was supplemented with 10 ng/mL of bFGF for hAC expansion. Basic medium was further supplemented with 1 mg/mL of insulin and 1 mg/mL of ascorbic acid, when hAC were seeded onto the PCL NFM (differentiation medium). These supplements enhanced extracellular matrix (ECM) deposition by the cultured hACs.

Human articular chondrocytes where used at passage 4. Expansion medium was changed every 2 days until the cells reached a confluence of 90%. The cells were harvested and seeded onto both the activated NFM, as well the NFM with the TNF-α antibody immobilized using the differentiation medium.

#### 2.6.2. Seeding onto NFMs

Electrospun PCL NFMs were sterilized by UV-O treatment. All the surface functionalization and TNF-α antibody immobilization steps were performed in sterile conditions. For the biofunctionalized NFMs, antibody immobilization was performed overnight, and after the BSA blocking step, cell seeding was performed. Activated and biofunctionalized electrospun NFMs were used as controls.

Confluent hACs were detached from the culture flask using trypsin, counted on a hemocytometer and seeded at a density of 200.000 cells/NFM. Seeding was performed using the droplet method, using a 50 µL drop of cell suspension on all sample groups in 24-well plates. The plates were placed at 37 °C and 5% CO_2_ over 4 hours. After cell attachment to the NFMs, 1 mL of expansion medium was added to each well. Afterwards, the medium was changed into differentiation medium. The seeding was performed in three independent experiments. For each independent experiment, constructs were cultured for 1, 14, 21 and 28 days under static conditions and collected at each time point for quantification of cell viability, DNA, total protein analysis, as well as GAGs deposition. Cell morphology was evaluated by SEM.

#### 2.6.3. DNA Quantification

DNA quantification was assessed using Quant-iT™ PicoGreen^®^ dsDNA Reagent and Kit (Life Technologies, Eugene, OR, USA), according to the manufacturer´s instructions. Triplicates of each condition collected at 1, 14, 21 and 28 days were evaluated. The specimens were collected, 1 mL of sterile distilled water was added, and then samples were stored at −80 °C until further analysis. Prior quantification, the samples were defrosted and sonicated for 15 min. To extrapolate the DNA values for each sample, a set of standards were prepared with concentrations ranging from 0 to 1.5 μg/mL. The fluorescence of each sample was measured on an opaque 96-well plate using an excitation of 485/20 nm and an emission of 528/20 nm, using a microplate reader (Synergy HT, BioTek, Winooski, VT, USA).

#### 2.6.4. Total Protein Synthesis Quantification

Total protein was performed using Micro BCA™ Protein Assay Kit (ThermoFisher Scientific, Rockford, IL, USA) according to the manufacturer´s instructions. Triplicates of each condition collected at 1, 14, 21, and 28 days were evaluated. The samples were collected as described in Section 2.6.3. A standard curve was prepared ranging from 0 to 200 μg/mL. The absorbance of each sample was measured at 562 nm using a microplate reader (Synergy HT, BioTek, Winooski, VT, USA).

#### 2.6.5. Glycosaminoglycan (GAG) Quantification

GAG quantification was performed using papain digestion. Triplicates of each condition collected at each previously established time point were tested. The samples were collected and stored in eppendorf tubes at −80 °C until further analysis. Digestion solution was prepared by adding papain (Sigma Aldrich, Saint Louis, MO, USA) and *N*-acetyl cysteine (Sigma Aldrich) at concentrations of 0.05% and 0.096%, respectively, to 50 mL of digestion buffer (200 mM of phosphate buffer containing 1 mM EDTA (Sigma Aldrich), pH 6.8). Each specimen was incubated with 600 μL of digestion buffer, overnight at 60 °C. After a 10-min centrifugation at 1300 rpm, the supernatant was collected. Dimethymethylene Blue (DMB) stock solution was prepared dissolving 16 mg of DMB powder in 900 mL of distilled water containing 3.04 g of glycine and 2.73 g of NaCl. pH was adjusted to 3.0 with HCl and the volume adjusted to 1 L. The solution was stored at RT covered by aluminum foil. Chondroitin sulphate (Sigma, C8529) solution was prepared in water in a 5 mg/mL stock solution and kept refrigerated. Standards were prepared from serial dilutions of this solution. Three samples per condition were considered per time point, and the absorbance of each sample was measured at 530 nm using a microplate reader.

#### 2.6.6. Histological Analysis

Samples were collected at the end of the experiment and processed for histology. Samples were then fixed in 10% neutral-buffered formalin and then kept at 4 °C until the staining procedures. Alcian Blue staining was performed by rinsing the samples in 3% acetic acid, keeping them in 1% alcian blue solution (Sigma, A-3157) for 1 h. After that, the stain was poured off and sections were washed with water, let to dry, and then rinsed in absolute alcohol, cleared in xylene, and mounted in Entellan rapid (Merck Millipore, Darmstadt, Germany, 1.07960.0500).

### 2.7. Statistical Analysis

Statistical analysis of values related with antibody immobilization, TNF-α capturing profiles and biological studies was performed using IBM SPSS software (version 21; SPSS Inc., Armonk, NY, USA). Firstly, a Shapiro-Wilk test was used to ascertain the assumption of data normality. For antibody immobilization, protein capture and biological assays, the results showed that the data was not following a normal distribution. P values lower than 0.01 were considered statistically significant in the analysis of the results. A Kruskal-Wallis test was also performed for the comparison of more than two independent groups of samples for one variable.

## 3. Results and Discussion

### 3.1. Antibody Immobilization Efficiency

**Figure 1:**
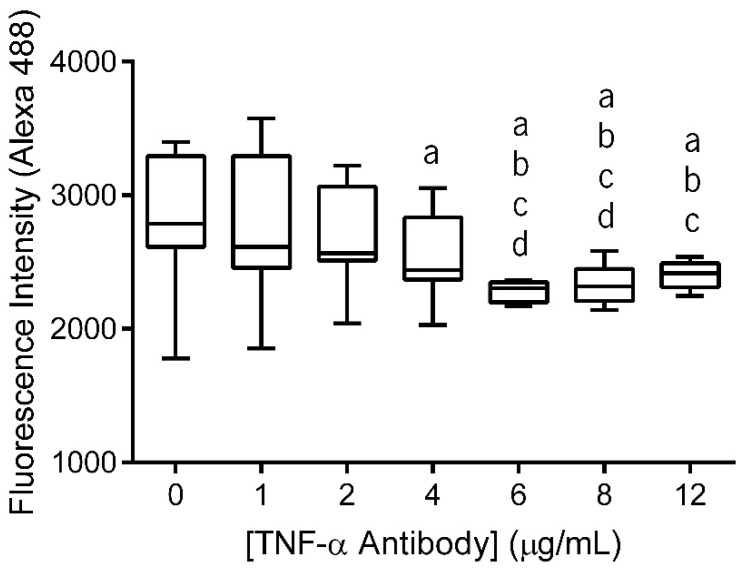
Box plot of anti-TNF-α antibody immobilization at concentrations ranging from 0 to 12 µg/mL. Data were analysed by nonparametric way of a Kruskal-Wallis test followed by Tukey’s HSD test: (**a**) denotes significant differences compared to concentration 0, (**b**) denotes significant differences compared to concentration 1, (**c**) denotes significant differences compared to concentration 2, and (**d**) denotes significant differences compared to concentration 4.

The primary antibody (anti-TNF-α antibody) immobilization at the surface of electrospun nanofiber meshes (NFMs) was performed in a wide range of concentrations (0–12 µg/mL) to determine the maximum antibody immobilization capacity. The fluorescence of the unbound secondary antibody was measured and the values were plotted against the various primary antibody concentrations (Figure 1). As this is an indirect quantification method, higher fluorescence values of the unbound secondary antibody correspond to lower concentrations of anti-TNF-α primary antibody immobilization. Moreover, when the substrate is saturated, the fluorescence values of unbound secondary antibody reach a plateau, despite the increment on antibody concentration.

The statistical analysis showed that the immobilized primary antibody, with concentrations above 4 μg/mL, displayed significantly lower values of fluorescence intensity than 0 μg/mL (*p* < 0.01). Concentrations of 6 μg/mL and 8 μg/mL exhibited significantly lower values of fluorescence intensity when compared to all lower concentrations (*p* < 0.001). Moreover, these two concentration values did not display differences between themselves. Finally, when the antibody was immobilized at a concentration of 12 μg/mL, the fluorescence intensity of secondary antibody did not present significant differences when compared to the fluorescence intensity of NFMs with primary antibody immobilized at concentrations above 2 µg/mL (*p* < 0.01). Therefore, primary antibody immobilization at the surface of the nanofibrous substrate reached a maximum concentration of 6 µg/mL, since no statistically significant differences were found between the concentrations above. This value is within the range of concentrations systemically administrated in clinical practice using Infliximab (3–10 mg/kg or 3–10 µL/mL, considering water as body mass) [52].

### 3.2. Spatial Distribution of the Antibody at the Surface of Electrospun Nanofibers

**Figure 2:**
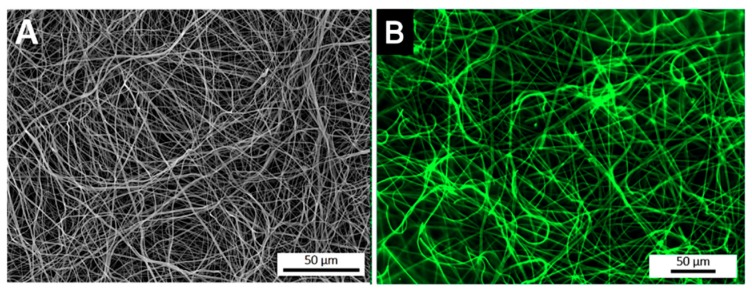
SEM micrograph of a biofunctionalized NFM (**A**). Fluorescence micrograph of a NFM with the TNF-α antibody immobilized at the maximum concentration (6 µg/mL) (**B**).

After the optimization of the anti-TNF-α antibody immobilization, its spatial distribution at the surface of electrospun NFMs was evaluated by fluorescence microscopy (Figure 2). The fluorescence images display a random mesh-like arrangement comparable to the typical morphology of the electrospun NFM (Figure 2A), showing that the antibody immobilization was successfully performed and uniformly distributed along the surface of the nanofibers (Figure 2B). To ensure that the secondary antibody was only bound to the immobilized primary antibody, a control experiment was defined, in which all biofunctionalization steps were performed, except for incubation with the primary antibody. The absence of a fluorescent signal proves that Alexa Fluor 488^®^ secondary antibody was not immobilized at the surface of activated and functionalized NFMs (data not shown), confirming the specific bond between the primary and secondary antibodies.

### 3.3. Quantification of Available Amine Groups

Antibody immobilization was achieved by the amine groups inserted at the nanofibers’ surface, which provides binding sites. Consequently, the availability of this functional group influences directly the primary antibody immobilization. To confirm the success of aminolysis functionalization, as well as to determine if the binding points were enough for primary antibody immobilization, quantification of available amine groups was performed. For this, SH groups were inserted at the surface of the aminolysis-treated NFMs and determined by the Ellman’s reagent method [49]. Table 1 presents SH groups quantification at the surface of bare, UV-O irradiated, aminolysis-treated, and fully biofunctionalized (with primary antibody immobilized) NFMs. The concentration of free SH groups at the surface of electrospun NFMs is at its maximum value for the aminolysis-treated NFM, whereas the bare NFM presents the minimum value, as expected. These values are in agreement with the ones described in the literature [49] and confirm that the insertion of amine groups was successful. Moreover, it was also proved that the amine groups inserted at the surface of activated NFM were enough for primary antibody immobilization, since free amine groups were still present. Indeed, only around 48% of the inserted amine groups were used in the immobilization of the TNF-α antibody at 6 µg/mL.

### 3.4. Quantification of Captured TNF-α

The ultimate goal of this work was to engineer a system that could capture TNF-α. Accordingly, after the primary antibody immobilization optimization, the TNF-α capturing capacity of the biofunctionalized substrate was assessed. A TNF-α rich conditioned culture medium from stimulated monocyte-derived macrophages was used over two distinct time intervals: 3 days representing the half-life of the anti-TNF-α antibody and 15 days representing the time interval of anti-TNF-α antibody administration in the clinic. Four conditions were tested and compared: (1) the biofunctionalized NFM (with primary antibody); (2) a positive control with the soluble form of the anti-TNF-α; (3) a negative control with UV-O activated NFM; and (4) the conditioned culture medium alone. The quantification of TNF-α levels from the conditioned culture medium (condition 4) was used as a baseline, eliminating the quantification of degraded TNF-α but not captured. Therefore, to guarantee the values’ accuracy and reproducibility between experiments, the values herein presented do not correspond to the ones quantified by ELISA, but just to the concentration of the TNF-α captured.

#### 3.4.1. TNF-α Capturing during 3 days

**Figure 3:**
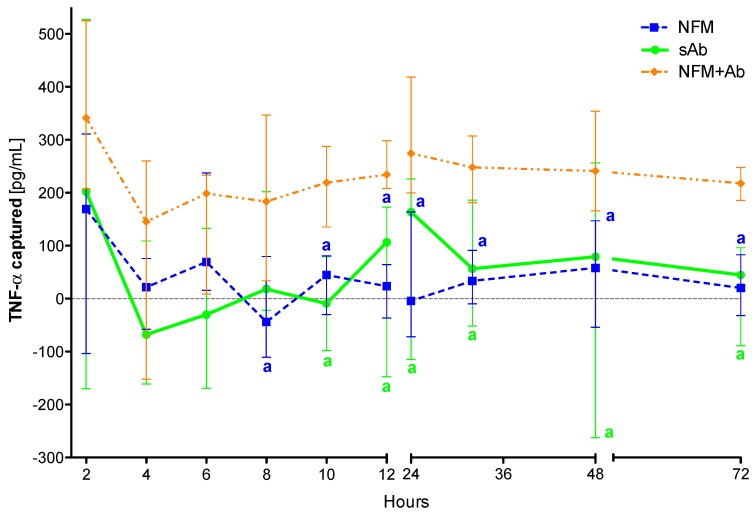
TNF-α captured (median and interquartile range) over 72 h. Data were analysed by nonparametric way of a Kruskal-Wallis test, followed by Tukey’s HSD test: (**a**) denotes significant differences compared to immobilized anti-TNF-α at the surface of electrospun NFM (condition NFM + Ab).

The proposed system comprising anti-TNF-α immobilized at the surface of NFMs (NFM + Ab) was found to capture soluble TNF-α in significant amounts after 8 hours of incubation when compared to the UV-O activated NFM (NFM) (*p* < 0.01) (Figure 3). This observation was also valid when the biofunctionalized substrate was compared to the soluble antibody (sAb), as the NFM + Ab exhibited significantly higher cytokine clearance for time points above the 10th hour of incubation (*p* < 0.01). In addition, no significant differences were found between the sAb and the control condition UV-O activated NFM (NFM).

The initial efficiency of the activated NFM over the TNF-α levels at early time points might be justified by the presence of free binding sites (such as the available amine groups quantified in Section 2.4) that unspecifically immobilize/adsorb proteins present in the conditioned medium, including TNF-α. Nevertheless, the capturing levels in this condition stabilize around zero for time points above 6 hours, unlike the biofunctionalized NFM + Ab, which maintains the capturing ability until the last time point. Likewise, the positive control sAb has a similar biological effect to the NFM + Ab testing condition during the first 10 hours. The sAb capturing ability decreases after this time point and becomes comparable to the NFM negative control condition. These results show that the immobilized form of anti-TNF-α is more stable and has longer action times, when compared to the non-immobilized one (i.e., sAb). However, this decrease might be partially justified by the gradual clearance of the soluble anti-TNF-α from the conditioned medium along the samples collection. It is envisioned that this decrease of sAb mimics its clearance in a living system, since the circulating antibody has higher propensity to be cleared by biological means [41]. Even if the sAb was locally administered, part of it would escape the articular cavity and enter into circulation, suffering also the clearance phenomena.

#### 3.4.2. TNF-α Capturing during 15 Days

**Figure 4:**
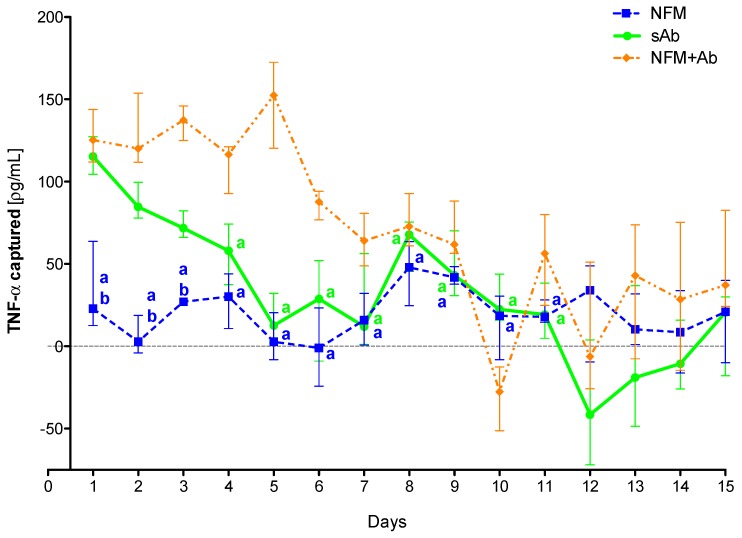
TNF-α captured (median and interquartile range) over 15 days. Data were analyzed by nonparametric way of a Kruskal-Wallis test, followed by Tukey’s HSD test: (**a**) denotes significant differences compared to immobilized anti-TNF at the surface of electrospun NFM (condition NFM + Ab); and (**b**) denotes significant differences compared to soluble anti-TNF (condition sAb).

To confirm the efficiency of the primary antibody immobilized at the NFMs’ surface to clear TNF-α from the conditioned medium over time, as well as to evaluate the action time of the immobilized antibody, an extended assay (15 days) was performed. Experimental results show that the sAb only has a biological effect for the first 3 days, since from the fourth time point on, it does not present significant differences when compared to the UV-O activated NFM (Figure 4). The NFM + Ab has a significantly higher antigen-neutralizing activity ranging from the 2nd day until the 11th day when compared to the sAb condition. Moreover, the biofunctionalized NFM + Ab does not have a significant biological effect from the 11th day on, as no significant differences were found when compared to the control NFM.

These results confirm the initial hypothesis that the immobilization of the primary antibody at the NFMs’ surface had a longer effect than the soluble antibody. This longer effect might be supported by the increased stability that the antibody immobilization provides [53]. Moreover, the physical attachment prevents the antibody clearance from the system by biological means. As each NFM has a high specific surface area, it allows the immobilization of high concentrations of primary antibody, requiring a small amount of material for the development of an effective cytokine capture system. Moreover, as the NFMs are made of polycaprolactone (PCL), a well-known biodegradable polymer, these membranes will be completely degraded within a maximum period of 2 years. Thus, the degradation time ensures that the captured TNF-α, as well as the attached antibody, may be slowly degraded/metabolized by biological processes and that there is no abrupt release of these molecules to the exterior of the synovial cavity.

### 3.5. Biologic Assays

**Figure 5:**
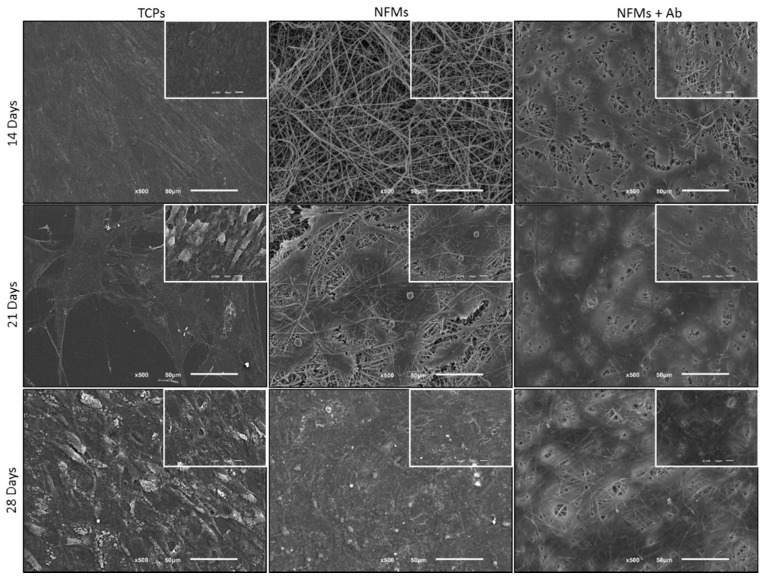
SEM micrographs of hACs cultures on tissue culture polystyrene (TCPs), UV-O treated electrospun PCL NFM (NFMs) and anti-TNF-α immobilized at the NFMs’ surface (NFM + Ab) for 14, 21 and 28 days.

After the successful assembly of the TNF-capturing system, we then tested it in contact with arthritic joint cells. Therefore, the toxicity of the biofunctionalized NFMs was assessed by culturing them with human articular chondrocytes (hACs). These cells were isolated from diseased knee arthroplasties, thus presenting a phenotype that is associated with RA and osteoarthritis pathologies, where inflammation was present. Different biological assays were conducted to assess the hACs morphology, proliferation, total protein, and glycosaminoglycans (GAGs) synthesis. hACs were cultured at the surface of three different substrates: (1) anti-TNF-α antibody immobilized at the surface of NFMs (NFM + Ab), (2) UV-O activated NFM (NFM) and (3) tissue culture polystyrene (TCPs) as positive control. The influence of the anti-TNF-α immobilization at the surface of NFMs over hACs morphology was assessed by SEM. Micrographs (Figure 5) showed that hACs maintained their round-shape morphology, typical of these chondrocytic cells.

**Figure 6:**
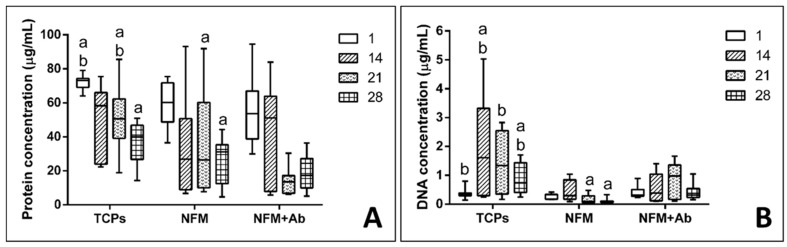
Box plot of human articular chondrocytes proliferation (**A**) and protein synthesis (**B**) when cultured on tissue culture polystyrene (TCPs), UV-O treated electrospun PCL NFM (NFM) and anti-TNF-α immobilized at the NFMs’ surface (NFM + Ab). Data were analysed by nonparametric way of a Kruskal-Wallis test, followed by Tukey’s HSD test: (**a**) denotes significant differences compared to NFM + Ab and (**b**) denotes significant differences compared to NFM.

Chondrocytes proliferation was also evaluated by quantifying the dsDNA concentration. Experimental results showed that the TCP’s condition displayed a significantly higher DNA concentration, for 14 and 28 days of culture, when compared to all other culture conditions (*p* < 0.001) (Figure 6A). Furthermore, on the 1st and 21st days of hACs culture, the control condition (TCPs) present similar proliferation to the NFM + Ab testing condition. Finally, the NFM condition presented a significantly lower cell proliferation at 21 and 28 days of culture, when compared to the NFM + AB condition (*p* < 0.001). Taken together, these results indicate that the immobilized anti-TNF-α may inhibit chondrocytes growth by mimicking an inflammatory environment, where cells would respond by limiting their growth and proliferation. This effect is not negative, since our primary goal was not to promote an extensive cell proliferation, but merely to assess the device’s potential cell toxicity. Nevertheless, hACs keep proliferating, even if at a lower rate when compared to the controls. Indeed, IL1-β and TNF-α have been shown to inhibit the migratory potential of chondrogenic progenitor cells in osteoarthritic cartilage [54]. Furthermore, OA-derived hACs cultures have been reported to excrete around 3000 pm/µL of TNF-α [55], thus these cells have mechanisms to respond to high concentrations of this cytokine in the surrounding environment.

Quantification of total protein synthesis is presented in Figure 6B. Experimental data showed that TCP’s control condition displayed significantly higher concentration values than all the other testing conditions for the 1st and 21st days of culture (*p* < 0.01). On the other hand, the NFM + Ab testing condition displayed a significantly lower protein concentration when compared to all other culture conditions for 28 days of culture (*p* < 0.01). At 14 days of hACs culture, no significant differences between conditions were found (*p* = 0.148). The protein synthesis in the NFM + Ab condition was lower for longer culture periods, which is possibly due to the extracellular accumulation of proteins by hACs, rather than the intracellular synthesizing rate.

**Figure 7:**
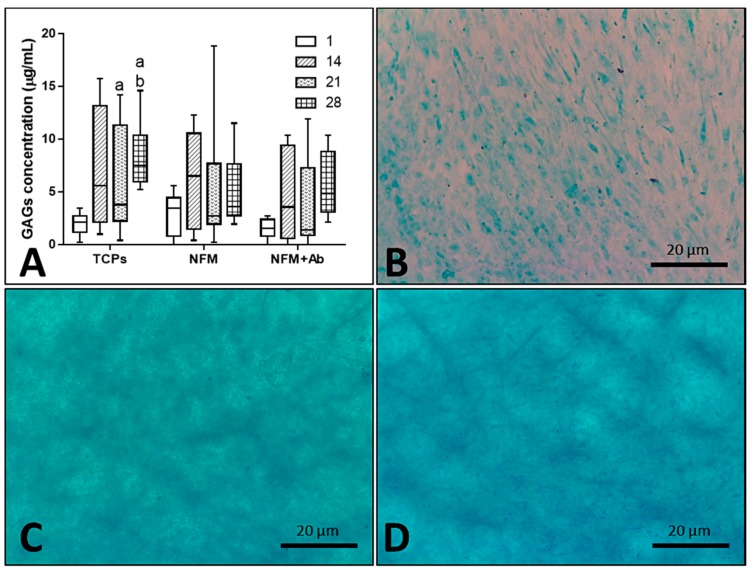
Blox plot of human articular chondrocytes GAGs accumulation when cultured on tissue culture polystyrene (TCPs), UV-O treated electrospun PCL NFM (NFM) and anti-TNF-α immobilized at the NFMs’ surface (NFM + Ab) (**A**). Data were analysed by nonparametric way of a Kruskal-Wallis test, followed by Tukey’s HSD test: (**a**) denotes significant differences compared to NFM + Ab and (**b**) denotes significant differences compared to NFM. Alcian blue staining evidencing sulphated proteoglycans deposition in samples from TCPs (**B**), NFMs (**C**), and NFM + Ab (**D**) on the 28^th^ day of the experiment.

Furthermore, the impact of the anti-TNF-α antibody immobilization at the surface of NFMs on GAG production and accumulation (Figure 7A) by hACs was also assessed. No significant differences were found between all culture conditions for the 1st (*p* = 0.186) and 14th days (*p* = 0.088) of the experiment. On the 21st day, the NFM + Ab testing condition displayed significantly lower GAG concentrations than control condition TCPs (*p* < 0.01). Moreover, on the 28th day, TCPs presented significant higher GAGs concentration than all other culture conditions (*p* < 0.01). Concerning the GAGs production by hACs cultured onto the NFM + Ab, the obtained results confirm the evidences found in the intracellular protein synthesis. There is a decrease in the total protein concentration values for longer time points, with a concomitant increase of GAGs production for these same periods of time, indicating that protein synthesis was being directed by the GAGs production. This increase of GAGs concentration for late time points of the experiment are in accordance to our hypothesis, as they will protect chondrocytes against the inflammatory environment.

As it can be observed in Figure 7B–D, sulphated proteoglycans, an important component of articular cartilage ECM, were detected on all culture conditions by alcian blue staining. Furthermore, these observations are reassured by the quantification of GAGs presented in the box plot of Figure 7A. Altogether, these results on intra- and extra-cellular protein synthesis confirmed that hACs are not only able to proliferate and maintain their typical round morphology, but are also capable of performing their metabolic functions properly, in the presence of the NFM + Ab testing system. With this study, we validated the use of this TNF-α capturing system nearby cartilaginous structures. There is, indeed, an influence on these parameters when cells are cultured onto NFM + Ab nanofibers, but it is not enough to restrain human articular chondrocytes functions. In fact, it has been described that the blockade of TNF-α by Infliximab treatment, during fracture healing, leads to an increased callus size. This increase was accompanied by an increase in cartilage tissue [56].

## 4. Conclusions

The present work proposes the use of nanofibrous meshes (NFM) functionalized with a neutralizing anti-TNF-α antibody, for the selective clearance of TNF-α. From a spectrum of primary antibody concentrations, 6 µg/mL was determined as the maximum immobilization capacity of a UV-O activated and aminolysis-functionalized NFM. Experimental results demonstrated the bioactivity of the developed biofunctional substrate on clearing TNF-α from conditioned culture medium of macrophages and a longer time of action when compared to the circulating/soluble antibody. Furthermore, cell biology data showed that the immobilized anti-TNF-α antibody exhibited no cytotoxicity over human articular chondrocytes and that these cells were able to maintain their metabolic functions in the presence of the TNF-capturing system. In conclusion, we herein describe an effective system for local clearance of TNF-α, which is particularly relevant in a RA scenario, but adaptable to other autoimmune inflammatory conditions.

## 5. Patents

Martins A, Oliveira C, Reis RL, Neves NM. Polymeric substrates with immobilized antibodies and method of production thereof. WO/2015/166414 A1, EP 3137906 A1, US 2017/0051050 A1. Priority date 28/04/2014.

## Figures and Tables

**Table 1:** Quantification of the free SH groups at the surface of untreated, activated, aminolys-treated, and biofunctionalized NFMs.

Condition	SH/NH_2_ Groups [mol/cm]
Untreated	(3.54 ± 5.61) × 10^−9^
UV-O activated	(5.86 ± 1.12) × 10^−9^
Aminolysis-treatment	(17.9 ± 6.06) × 10^−9^
Immobilized Antibody	(8.65 ± 3.41) × 10^−9^
